# A case of COVID‐19 with superficial thrombophlebitis caused by an indwelling peripheral venous catheter

**DOI:** 10.1002/ccr3.4987

**Published:** 2021-10-23

**Authors:** Hiroaki Tachi, Midori Hanazawa, Takashi Matsuda, Kei Shimizu, Yusuke Yamamoto

**Affiliations:** ^1^ Department of Respiratory Medicine Hitachi General Hospital Hitachi, Ltd Hitachi Japan

**Keywords:** indwelling peripheral venous catheter, local hypercoagulable state, SARS‐CoV‐2, superficial thrombophlebitis, vascular endothelial cell damage

## Abstract

A local hypercoagulable state caused by SARS‐CoV‐2 and an indwelling peripheral venous catheter can lead to superficial thrombophlebitis. If the venous catheter is no longer needed during treatment for COVID‐19 it should be removed promptly.

## Graphical abstract

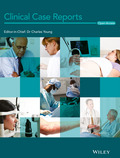

## 1 INTRODUCTION

A 53‐year‐old man hospitalized because of COVID‐19 developed non‐infectious superficial thrombophlebitis. The thrombus formed only in the vessel where the peripheral venous catheter was placed. This complication was considered to be caused by damage to vascular endothelial cells by SARS‐CoV‐2 and an indwelling catheter, leading to a local hypercoagulable state.

Coronavirus disease 2019 (COVID‐19) is caused by severe acute respiratory syndrome coronavirus 2 (SARS‐CoV‐2) infection, and cytokine storm and microthrombus formation affect the severity of the disease. COVID‐19 mainly affects the respiratory system, but it is often complicated by venous thromboembolism (VTE), such as pulmonary thromboembolism (PE) and deep vein thrombosis (DVT), due to a systemic hypercoagulable state. However, there are few reports of thrombus formation in peripheral veins resulting in superficial thrombophlebitis. On the contrary, indwelling peripheral venous catheters can cause catheter‐related bloodstream infections and venous thrombus formation, albeit less frequently. We report a rare case of COVID‐19 in which superficial thrombophlebitis developed due to thrombus formation only in the vessel in which the peripheral venous catheter was indwelled.

## 2 CASE PRESENTATION

**FIGURE 1:**
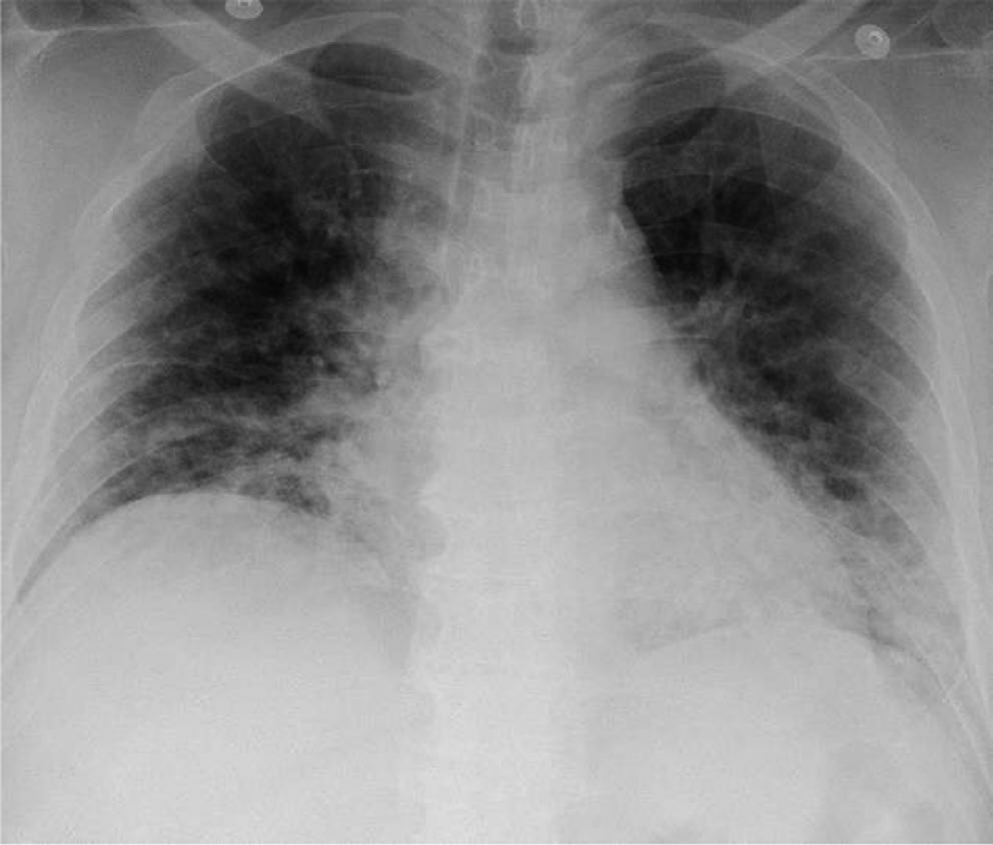
Chest X‐ray on admission to the referring hospital. Ground‐glass opacities and infiltrative shadows were observed in both lung fields

**FIGURE 2:**
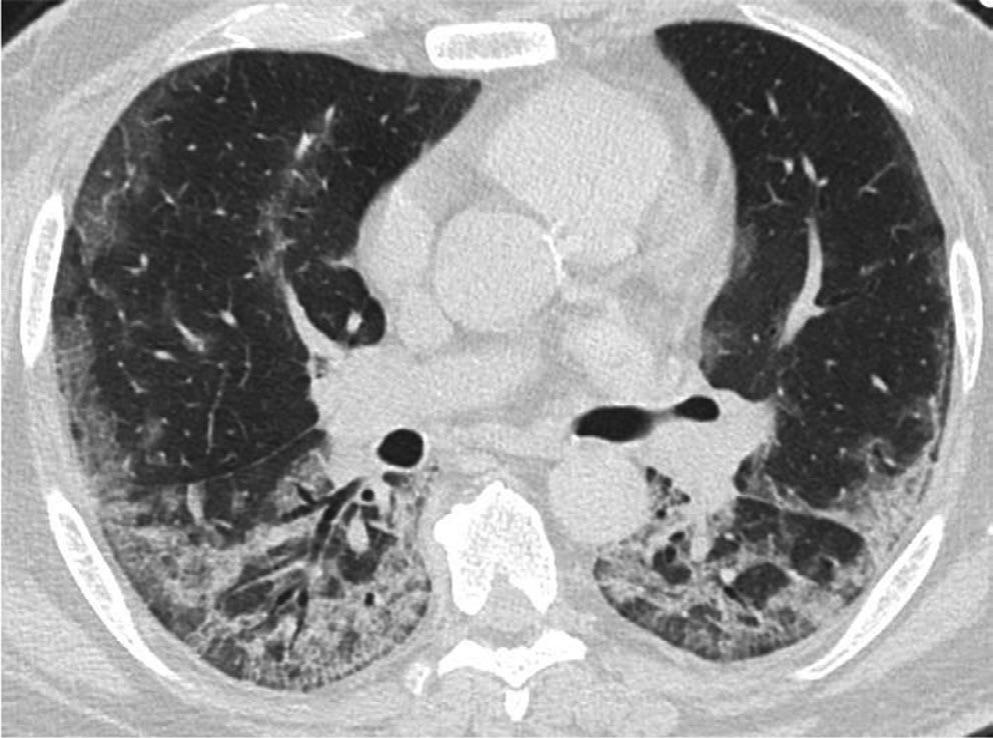
Chest computed tomography on admission to the referring hospital. Ground‐glass opacities and infiltrative shadows were observed in the lower lobes of the bilateral lungs with a subpleural predominance

In April 2021, on the day following a dinner party with four friends, a 53‐year‐old man presented with cough, anorexia, and general malaise, which exacerbated thereafter, and fever of 39°C was also observed. On the 16th day of onset, the patient visited his local physician who found infiltrative shadows in both lung fields on chest X‐ray (Figure 1). On the same day, he was referred to a general hospital, where SARS‐CoV‐2 was detected by nasopharyngeal swab, and he was admitted because of respiratory failure. Chest computed tomography demonstrated ground‐glass opacities and infiltrative shadows in the lower lobes of the bilateral lungs with a subpleural predominance (Figure 2), and he was diagnosed with severe COVID‐19. His background factors (risk of severe COVID‐19) included obesity (body mass index of 30), former smoker (30 pack‐years), and type 2 diabetes mellitus under treatment. There was no history of vaccination. Although a high‐flow nasal cannula (HFNC) was inserted, due to a deteriorating respiratory condition, he was intubated in the evening of the same day, and administered 6.6 mg of dexamethasone intravenously and enoxaparin sodium subcutaneously. However, on the 3rd day of hospitalization (18th day of onset), his respiratory condition did not improve. As extracorporeal membrane oxygenation (ECMO) was considered, he was transferred to our hospital on the same day. After admission to the intensive care unit (ICU), his treatment was continued and supine management was started (without remdesivir or tocilizumab). As the oxygen demand improved, he was extubated on the 6th day of transfer and HFNC was reintroduced, which was completed on the 10th day of transfer, and oxygen administration was completed on the 13th day of transfer. Dexamethasone was administered for 10 days, enoxaparin sodium was administered for 11 days, and the anticoagulant was changed to edoxaban.

**FIGURE 3:**
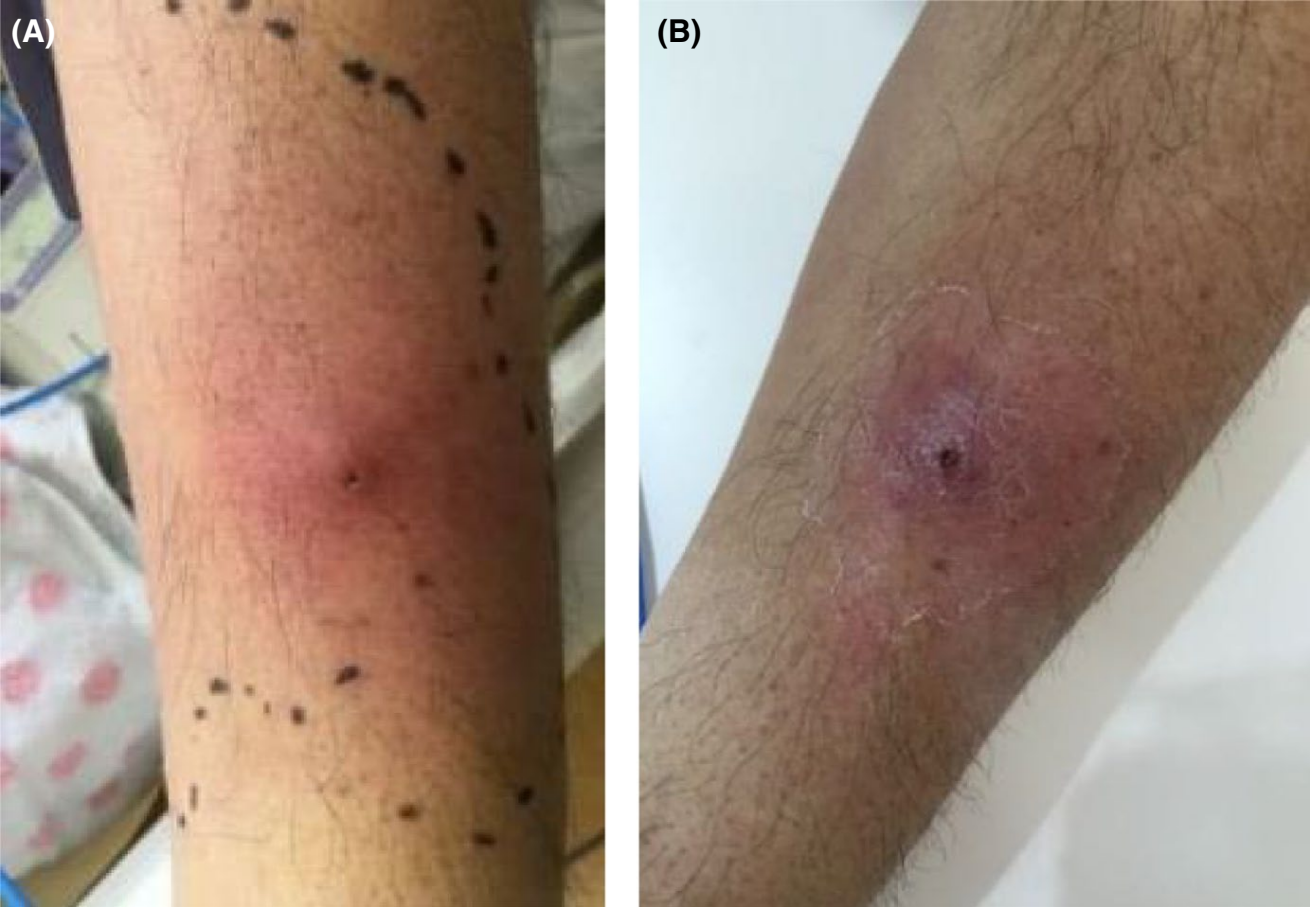
Image of the left forearm. (A) Redness and swelling were observed around the insertion site of the catheter in the cephalic vein on the 26th day of onset. (B) After removal of the catheter, the redness and swelling improved

**FIGURE 4:**
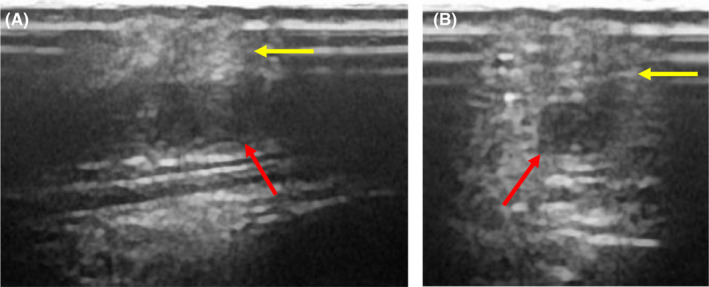
Vascular ultrasonography showed a thrombus in the lumen of the cephalic vein of the left forearm (red arrow), accompanied by inflammatory findings in adjacent tissues (yellow arrow). (A) Longitudinal view. (B) Transverse view

On the 9th day of transfer to our hospital (26th day of onset), the peripheral venous catheter placed in the cephalic vein of the left forearm was removed. The next day, redness, swelling, and pain were observed around the site of catheter insertion (Figure 3). Blood tests revealed increased CRP (2.58 mg/dl on day 26 to 9.28 mg/dl on day 29). Although he did not have fever, catheter‐related bloodstream infection (CRBSI) was suspected based on skin findings. Vancomycin (VCM) was started, but blood cultures were negative one week later and CRBSI was also negative, leading to the discontinuation of VCM. Vascular ultrasonography demonstrated a thrombus in the cephalic vein of the left forearm (Figure 4A,4B), accompanied by inflammation in the surrounding tissues, which was diagnosed as superficial thrombophlebitis. D‐dimer levels peaked on the 27th day of onset and decreased, and CRP and skin findings improved (Figure 3B). No evidence of DVT in the lower limbs was found on ultrasonography, but PE was not evaluated because contrast‐enhanced CT was not performed. Although we did not consider infectious disease, cellulitis was unable to be excluded; therefore, cefditoren pivoxil was started the day after completing VCM. After confirming improvement in the skin findings on the left forearm, both edoxaban and cefditoren pivoxil were discontinued after 2 weeks. The patient was discharged on the 23rd day of transfer (40th day of onset) with good progress.

## 3 DISCUSSION

To the best of our knowledge, this is the first case report of COVID‐19 with superficial thrombophlebitis mainly related to an indwelling peripheral venous catheter. There are few reports of superficial thrombophlebitis associated with COVID‐19. Demirbas et al.^1^ reported only one case, but it was not in a peripheral vein with an indwelling catheter. There is a report on VTE from northern Italy^2^ in which superficial thrombophlebitis was observed in 7 of 101 COVID‐19 patients who underwent ultrasonography, and only one case was in the cephalic vein as in the present case. However, there was no mention of whether it was an indwelling catheter, making the present case valuable.

In February 2020, SARS‐CoV‐2 infection was named COVID‐19 by the WHO and it has since become a worldwide pandemic.^3^ COVID‐19 mainly affects the respiratory system, causing a variety of symptoms ranging from mild flu‐like symptoms to severe pneumonia, but effects on organs besides the respiratory system have also been reported.^4^ Venous thrombosis, such as PE and DVT, has been reported frequently, especially in patients requiring ICU management.^5^, ^6^, ^7^ Cytokine storm induced by SARS‐CoV‐2 infection is known to cause widespread microvascular and macrovascular thrombosis and organ failure.^8^ A report of lungs autopsied after COVID‐19^9^ revealed severe vascular endothelial cell damage, resulting in the occlusion of alveolar capillaries by microthrombus formation. Angiotensin‐converting enzyme 2 (ACE2), a receptor for SARS‐CoV‐2, is highly expressed in the lungs, which are considered a target organ, but there is a report that ACE2 is also expressed in the vascular system, including endothelial cells and vascular smooth muscle cells.^10^ In this case, although the patient was receiving anticoagulant therapy, the excessive inflammation caused by SARS‐CoV‐2 damaged vascular endothelial cells in peripheral veins via ACE2 and exacerbated the local hypercoagulable state, resulting in thrombus formation and thrombophlebitis.

Superficial thrombophlebitis is a disease in which a blood clot forms and obstructs a peripheral vein, causing inflammation of the vein, surrounding tissue, and skin. Most cases of thrombophlebitis develop in the veins of the lower limbs,^11^ but it can also develop in the upper limbs due to endogenous factors, such as Buerger's disease,^12^ which damages the blood vessels themselves, or due to exogenous factors such as trauma or catheterization.^13^ The thrombophlebitis in this case had no relationship with endogenous disease or trauma. Although a systemic search for thrombus was not performed, DVT was not observed, and as it was considered to be local hypercoagulability rather than systemic because there were no findings suggestive of thrombosis in other organs, the catheter placed in the peripheral vein may have damaged the vascular endothelial cells, leading to thrombus formation and superficial thrombophlebitis.

Other factors can also lead to thrombus formation and phlebitis. Thrombosis due to central venous catheters is associated with CRBSI.^14^ In addition, Hernandez et al.^15^ reported that the rate of thrombus formation was three times higher in the group with CRBSI than in that without CRBSI in a study of hemodialysis catheters. This case was initially thought to be CRBSI because of the skin findings, increased inflammatory reaction on blood testing, and intravenous thrombus, but as CRBSI is rarely observed with peripheral venous catheters (0.2 cases per 100 catheters^16^) and the blood culture was initially negative (catheter tip culture test was not performed), CRBSI was excluded. Peripheral venous catheters are left in place with a heparin lock, but it has been reported that heparin promotes the adhesion of coagulase‐negative Staphylococcus (CNS) to the catheter^17^ and that regular heparin use, even at doses as low as 250–500 units per day, can cause thromboembolism.^18^ ^)^ However, in this case, continuous intravenous infusion of Ringer's acetate solution, furosemide, and dexmedetomidine was performed, and heparin was not administered, excluding thrombus formation due to heparin. The CDC recommends not replacing peripheral catheters more frequently than every 72–96 h to reduce risk of infection and phlebitis in adults.^19^ In this case, the catheter was placed for less than 3 days (the date of catheter placement was unknown, but our ICU has a rule of regular replacement every 3 days), and the skin findings were unremarkable from the time of catheter insertion to the time of removal. Anticoagulant therapy against COVID‐19 may act as a prophylactic against thrombus formation due to the indwelling catheter. Therefore, preventing the superficial thrombophlebitis in this case may have been difficult despite regular skin observation, adherence to proper catheterization duration, and prophylactic anticoagulation.

We reported a valuable case of COVID‐19 complicated by thrombophlebitis in the cephalic vein of the left forearm. In this case, superficial thrombophlebitis developed even while the patient was receiving anticoagulant therapy for COVID‐19, suggesting that catheter placement in peripheral veins during COVID‐19 treatment is a risk factor for thrombus formation. Although securing the peripheral venous route for COVID‐19 is a frequently performed procedure, there have been few reports of superficial thrombophlebitis and the reason for its development in the present case is unknown. Tager et al.^20^ reported that the risk of phlebitis increases with longer indwelling time of up to 6 days after catheter placement; therefore, prompt removal should be considered when the catheter is no longer needed, especially during treatment for COVID‐19.

## CONFLICTS OF INTEREST

None of the authors have any conflicts of interest.

## AUTHOR CONTRIBUTIONS

HT treated the patient and wrote the manuscript. All authors read and approved the final manuscript.

## ETHICAL APPROVAL

Approval by the research ethics committee was not required for the publication of this case report.

## CONSENT

Informed consent was received from the patient.

## Data Availability

Data sharing is not applicable to this article as no datasets were generated or analyzed during the current study.
